# Changes in Body Weight and Psychotropic Drugs: A Systematic Synthesis of the Literature

**DOI:** 10.1371/journal.pone.0036889

**Published:** 2012-06-15

**Authors:** Robert Dent, Angelique Blackmore, Joan Peterson, Rami Habib, Gary Peter Kay, Alan Gervais, Valerie Taylor, George Wells

**Affiliations:** 1 Weight Management Clinic, Ottawa Hospital, Ontario, Canada; 2 Ottawa Health Research Institute, Ottawa, Ontario, Canada; 3 Department of Psychiatry, University of Ottawa, Ottawa, Ontario, Canada; 4 University of Toronto, Toronto, Ontario, Canada; 5 Cardiovascular Research Methods Centre, University of Ottawa Heart Institute, Ottawa, Ontario, Canada; University of Manitoba, Canada

## Abstract

**Introduction:**

Psychotropic medication use is associated with weight gain. While there are studies and reviews comparing weight gain for psychotropics within some classes, clinicians frequently use drugs from different classes to treat psychiatric disorders.

**Objective:**

To undertake a systematic review of all classes of psychotropics to provide an all encompassing evidence-based tool that would allow clinicians to determine the risks of weight gain in making both intra-class and interclass choices of psychotropics.

**Methodology and Results:**

We developed a novel hierarchical search strategy that made use of systematic reviews that were already available. When such evidence was not available we went on to evaluate randomly controlled trials, followed by cohort and other clinical trials, narrative reviews, and, where necessary, clinical opinion and anecdotal evidence. The data from the publication with the highest level of evidence based on our hierarchical classification was presented. Recommendations from an expert panel supplemented the evidence used to rank these drugs within their respective classes. Approximately 9500 articles were identified in our literature search of which 666 citations were retrieved. We were able to rank most of the psychotropics based on the available evidence and recommendations from subject matter experts. There were few discrepancies between published evidence and the expert panel in ranking these drugs.

**Conclusion:**

Potential for weight gain is an important consideration in choice of any psychotropic. This tool will help clinicians select psychotropics on a case-by-case basis in order to minimize the impact of weight gain when making both intra-class and interclass choices.

## Introduction

Weight Gain is associated with psychotropic medication use, and while particular attention has been paid to atypical antipsychotics, the typical antipsychotics, mood stabilizers, tricyclic antidepressants (TCA’s), certain serotonin selective reuptake inhibitors (SSRIs), and serotonin norepinephrine reuptake inhibitors (SNRIs) can cause weight gain as well. Because weight gain and obesity are often overlooked in patients [1], there can be a lack of follow-up to monitor for weight gain [2]–[7] or subsequent weight related co-morbidities [8].

Psychotropic-induced weight gain is an important cause of non-adherence to pharmacotherapy for antidepressant medications [9]–[14], for antipsychotic medications [15]–[22] and for lithium [23], [24] and has been cited by an expert consensus panel on adherence problems in serious and persistent mental illness [25], [26]. Non-adherence to prescribed medications places patients at a greatly increased risk of illness exacerbation and re-hospitalization. These costs are high [27], and were estimated to range from $1392 million to $1826 million in 2005 in the US for antipsychotics alone [28]. These issues are balanced by the therapeutic benefit of the psychiatric medication. The CATIE trial concluded that the superior efficacy of olanzapine might prevent discontinuation due to weight gain [29], [30]. This may suggest the potential for weight gain may be offset by effectiveness or lack of other adverse events.

Psychotropic-associated weight gain carries significant risk. As a consequence, the weight-related co-morbidities associated with these medications have been the most studied and we now have a plethora of evidence on glucose dysregulation [29], [31]–[41], increases in triglycerides [29], [41] and total cholesterol [29], [42] and hypertension. Fontaine [43] estimated that weight gain associated with this class of drugs contributed to an increase in mortality that offset the decreased risk of suicide with their use.

The adverse effects of long term weight gain have not escaped regulatory bodies. A number of clinical practice guidelines [4], [44], [45] and other studies [46]–[50] all recommend choosing psychotropics least likely to cause weight gain, or switching to those less likely to cause weight gain [51]–[53] if weight gain occurs. This is because the CATIE trial data does provide some evidence that patients who stayed on medications with high propensity to induce weight gain, showed greater weight gain than those who switched from these medications to other drugs that were less likely to cause weight gain [54].

There are studies and reviews comparing weight gain for psychotropics within classes for the atypical antipsychotics [29], typical antipsychotics [36] and antidepressants [55]. But clinicians frequently use drugs from many different classes to treat any one psychiatric disorder. Therefore, we saw a need for an all encompassing evidence-based tool that would allow clinicians to balance efficacy against the risks of weight gain in making both intra-class and interclass choices of psychotropics [45], [56]–[59].

Our primary objective was to consider weight change with psychotropic drugs in adults with psychiatric conditions comparing drugs to placebos or other psychotropics, more specifically, to answer the following questions:

1. Is a particular psychotropic weight-neutral or is it associated with weight gain or weight loss?
2. Can the weight gain be quantified?
3. What is the difference between the weight gain in drug-naïve patients and the weight gain in those already on psychotropics?
4. How does the psychotropic rank with respect to weight gain in its class?

Our secondary objective was to develop a clinical tool that would provide information on psychotropic-associated weight gain to allow clinicians to make informed choices with respect to this important side effect.

## Methods

While a Cochrane-style review is well suited for finding the weight gain potential of a single drug or even a class of psychotropics it becomes very cumbersome when seeking evidence for all classes of psychotropics. We therefore developed a hierarchical search strategy (Table 1) that made use of systematic reviews that were already available. When such evidence was not available we went on to evaluate clinical trials that were double blind and randomly controlled, followed by cohort and other clinical trials.

**Table 1:** The Hierarchical strategy for selection of reports.

Level of study	Description	Rules for selection
I	Systematic Review where weight change is the focus or a key word	Rate according to Amstar [97]; The minimum criteria for a systematic review would be a search in 2 electronic databases using a stated search strategy; Where such reviews exist, choose in descending order: the one with the highest rating and the most recent; If after 2 years of the chosen review, there is a study in category III, then it is reviewed to determine if it changes the outcome; If there are two reviews at level 1 or 2 of the same year, the one with the higher rating is chosen; If a systematic review contained only one randomized controlled trial (RCT) dealing with weight then it is accepted as a systematic review because it was felt that the process yielding negative results was important.
II	Systematic Review where weight change is not the focus but “side effects” or “adverse events” or “tolerability” are present in the key words or abstract.	Similar to above
III	RCTs where weight change is a key word	Duration >12 wks, n >50; Rate with SIGN 50 [98]; Where such studies exist as the highest level of evidence, choose the one with the highest methodological rating and the most recent and no other study; Where there is more than one RCT and there is disagreement, then chose the one with the highest rating and acknowledge that there is disagreement
IV	Cross-sectional or population studies where weight change is a key word	Duration >12 wks, n >50; Rate with SIGN 50 [98]; Where such studies exist as the highest level of evidence, choose in descending order, the one with the highest methodology rating according to SIGN 50 [98] and the most recent.
V	Narrative Review with weight change is a key word	Not graded; Only used if no other in levels I-IV available; If a narrative review contained only one RCT dealing with weight then that RCT would be put in category 4 and take precedence over the narrative review.
VI	Other evidence/clinical experience or studies that would be IV, or V where the n<50 or duration <12 wks	Not graded; Only used if no other in levels I-V available.

### Inclusion Criteria

Studies were included if they contained information about psychotropic drug use in patients with a psychiatric disease (anxiety disorder, depressive illness, psychosis) or related condition (chronic pain, fibromyalgia, chronic fatigue). The psychotropic medication must have been compared with a placebo or comparator drug, ideally for 12 or more weeks and reviews had to report on weight change.

### Exclusion Criteria

We excluded children (since normal growth would be a confounder to evaluating weight gain) and patients with ADHD (since many of the studies were done in children). We also excluded subpopulations that may not be able to express drug-induced weight gain, such as the elderly with dementia, those in controlled environments where they may not have free access to food, those with anorexia nervosa, bulimia nervosa, malignancies and HIV disease. Studies were also excluded where the study drug was added to multiple other psychotropics.

### The Literature Search

A medical librarian searched a number of databases (from their inception to April 2011) for articles where weight gain was designated as the outcome or key word (Ovid Medline search strategy Appendix S1, PsycINFO search strategy Appendix S2, CCTR, CDSR (coch), Dare Search Strategy Appendix S3, Embase search strategy Appendix S4.) These data bases were then searched again, using the same search strategy and key words for systematic reviews where weight gain was not a key word or designated outcome. The searches were limited to English only [60].

The literature search yielded almost 9500 reports. Two of four potential reviewers (RD, AB, JP, GK) screened the reports for eligibility according to the criteria in Table 1: on the basis of title, then abstract, and then full-text reviews. At the title review stage, any title selected by either reviewer was included in the abstract review.

### Assessment of Articles

Each study was given a number from I to VI based on the hierarchical classification of the study according to the pre-established criteria in Table 1. We used the AMSTAR scale, a reliable and valid 11-item checklist for evaluating systemic reviews to assess the methodological quality of the reviews chosen [61] and graded according to good (A), fair (B), and poor (C). The quality of each randomized controlled trial, cross-sectional or population study was assessed using the SIGN 50 assessment form and similarly graded [62]. The quality of evidence for change in weight for a particular drug in a trial was graded with a score of 1, 2, or 3 according to quality. Each study was scored independently by two out of four potential reviewers (AB, JP, RH, GK) and disagreements were resolved by consensus. The individual that did the scoring was never the same individual that did the initial reviewing of that article.

### Data Extraction and Building of a Database

For each article that met inclusion/exclusion criteria, quantitative data (actual weight gain in drug-naïve and non drug-naïve patients) of the study drug and its comparators was sought wherever possible. If there was no quantitative evidence, the study with the best qualitative evidence was obtained, whether or not the drug was associated with weight gain. The sources of funding, either direct (funding from a pharmaceutical manufacturer), indirect (where authors had research funds) or unknown/unfunded, psychiatric disease and the duration of study were also extracted. Three groups of studies were collected: those giving qualitative or quantitative information on a drug, those comparing drugs within a class and those reporting weight gain in drug-naïve patients.

### Ranking of Psychotropics that Reported Weight Gain

In order to rank the weight gain caused by psychotropics, we selected studies that dealt with multiple drugs. Because we were not aware of any study that included all psychotropic drugs within their respective class, we included all of the studies that qualified. The data on ranking was extracted from each article and placed in a separate table to allow a comparison of the change in weight caused by psychotropics.

### The Subject Matter Expert Panel

An expert panel was formed to review the rankings and to deal with any potential discrepancies between articles. The methodology for review and the membership were formulated by an epidemiologist (G.W.). This panel consisted of 6 members: 4 psychiatrists (C.M., G.K., V.T.,R.H.); 1 family physician (S.W.), whose practice was large and busy enough to include a large number of patients with psychiatric illnesses and 1 internist (J.S.). Secretarial assistance was provided by A.G. and R.D.

The mandate of this panel was to review the literature that was used in developing the ranking of the psychotropics and to provide comments on the rankings based on their clinical experience. When there was a disagreement in the rankings, or when the rankings were at variance from the clinicians’ experience, the panel was asked to re-examine the articles in detail and attempt to provide a rationale for the controversy. All controversies were noted, as was the corresponding rationale.

### Classification of Psychotropics and Presentation of Data

The recommendations from the panel were subsequently used to rank the drugs within their respective classes. We have used a common and largely mechanistic classification for the psychotropics [63]. There does not seem to be a standardized classification – often the term “second generation antipsychotics” is used rather than “atypical antipsychotics” [45]. Given the results of the review and the input of the expert panel, a table of weight analysis was constructed.

## Results

The screening for eligibility began by examining 3975 articles (Figure S1). They included systemic reviews, randomized controlled studies, cross-sectional or population studies, and narrative reviews where weight gain was the focus. Of these, 956 articles were requested for full text review. The second search of systemic reviews, where weight gain was not a key word or identified in the abstract, screened an additional 5500 articles. Of these, 957 were requested for full text review. A short list of 666 articles resulted.

The older classes of psychotropics yield very little information on weight gain. Ideally, the best ranking evidence for psychotropics would come from drug-naïve patients, but there was no ranking data available. We were only able to find data in drug-naïve patients for 7 antipsychotics olanzapine, chlorpromazine, clozapine, quetiapine, risperidone, aripiprazole and haloperidol. There were 14 articles that met our hierarchical search strategy to enable us to rank psychotropics. Two articles [60], [61] ranked the MAOIs (Table 2). Seven articles ranked the typical and atypical antipsychotics (Table 3). Five articles were classified as level I, three with good quality of evidence [62]–[64] and two provided evidence that was fair. Two articles were level III with good quality of evidence [29], [65].

**Table 2:** MAOI ranking (ranked from most to least weight gain).

MAOI	Author	Study Level	Quality of Evidence for the Drug	Funding Source	Effect on Weight
Phenelzine	Garland [64]	V	3	U	Qualitative weight gain only.
Isocarboxazid	Cantu [65]	V	3	U	Qualitative weight gain only
Tranylcypromine	Garland [64]	V	3	U	Significant weight gain not noted when compared with phenelzine

U  =  unfunded or unknown funding.

**Table 3:** Typical and atypical antipsychotic ranking and change in body weight (ranked from most to least weight gain).

Antidepressant	Author	Study Level	Quality of Study	Quality of Evidence for the Drug	Funding Source	Quantitative Weight Gain	Comments	Articles used for ranking
Clozapine	Bitter [99]	III	B	2	D	4.1±5.6 kg.	Olanzapine 3.3±5.3 kg over 18 wks, not significant between groups	[63]–[65], [75]
	Lieberman [29]	III	A	2	D	Drug Naïve 9.9 kg	Chlorpromazine mean weight gain at 52 weeks (6.5 kg). Not statistically significant.	Not ranked
Olanzapine	Komossa [100]	I	A	1	I	10X and 2.5X greater wt gain with olanzapine	vs amisulpride (2 studies, 26 & 24 weeks)	[29], [62], [63], [65], [75], [76], [100]
		I	A	1		6X greater wt gain with olanzapine	vs aripiprazole (1 study 26 weeks)	
		I	A	1		3 studies show greater wt gain with clozapine	vs clozapine (4 studies)	
		I	A	1		10X, 1.5X, 1.5X 2X, 4X, 1.8X, 2X greater wt gain with olanzapine	vs risperidone (7 studies: 78, 52, 52, 30, 52, 28, 22 wks)	
	Alvarez-Jiminez [62]	I	A	1	I	Drug Naive: 7.1–9.2 kg or 47–61%	10–12 weeks; 3 studies, up to 4 fold greater weight gain in drug naïve.	Not ranked
		I	A	1	I	10.2–15.4 kg or 80–100%	>9 mos:3 studies	
Olanzapine orally dissolving	Karagianis [101]	I	C	3	U	Drug Naïve: first episode psychosis: 3.3 kg wt gain.	First episode psychosis oral tablets: 6.4 kg in 6 weeks	Not ranked
Olanzapine IM	Canas [102]	I	B	1	D	Mean weight gain 1.4 kg, 28%.	Long term similar to oral olanzapine	Not ranked
Thioridazine	Fenton [103]	I	A	2	I	Wt gain >4.5 kg: 3/15	Wt. gain >4.5 kg 5/15 with Pimozide, 1/10 with Placebo. Only 1 study, 6 month duration	[65]
Chlorpromazine	Allison [65]	I	B	3	D	2.1 kg	10 weeks	[65]
	Lieberman [104]	III	A	2	D	Drug Naïve: mean weight gain 6.5 kg	clozapine mean weight gain at 52 weeks (9.9 kg). Not statistically significant.	Not ranked
Quetiapine	Komossa [64]	I	A	1	I	2x more gained >7% with clozapine;	vs clozapine	[29], [63], [64], [75], [76]
		I	A	1		Mean weight gains 2 to 8x greater with olanzapine	vs olanzapine (4 studies >12 weeks)	
		I	A	1		2 to 3x gain >7% initial weight with olanzapine	vs olanzapine (2 studies >12 weeks)	
		I	A	1		Equal (mean weight gains and % gaining >7%).	vs risperidone (7 studies, >12 weeks)	
		I	A	1		Greater with quetiapine (mean weight gain and % gaining >7%)	vs. ziprazidone	
	McEvoy [105]	III	A	1		Drug Naive : M: 4.3 kg or 20%; F : 2.1 kg or 6%;	72% remained in at 12 weeks.	Not ranked
						M: 6.9 kg or 11%; F: 2.9 kg or 4%	33% remained in at 52 weeks.	
Risperidone	Alvarez-Jiminez [62]	I	A	1	I	1–2.3 kg or 9–11%n (0.4–3.9 kg)	10–12 wks (>9 mos)	[29], [62]–[65], [75], [76]
	Alvarez-Jiminez [62]	I	A	1		Drug Naïve: 4.0–5.6 kg or 33–38%	5 studies 10–12 wks; up to 4 fold greater weight gain in drug naïve	Not ranked
						6.6–8.9 kg or 58%	3 studies >9 mos	
Risperidone injectable	Canas [102]	I	B	1	D	Mean 0.95 kg (range 0.4 to 1.9 kg); [mean of 3 kg (range 2–3.3 kg)].	3–6 mos [over 1 year]	Not ranked
Amisulpride	Komossa [106]	I	A	1	I			[63], [65], [75]
		I	A	1		14%	vs risperidone: 20% (26 weeks)	
		I	A	1		17%	vs ziprasidone: 8% (12 weeks)	
		I	A	1		8%: Mean weight gain 0.21 kg	vs olanzapine: 22% (26 weeks) Mean weight gain: 2.43 kg	
		I	A	1		Mean weight gain 1.6 kg	vs olanzapine: (24 weeks) Mean weight gain 3.9 kg	
		I	A	1		13%: Mean weight loss -1.37 kg	vs olanzapine: 36% (26 weeks) Mean weight gain 8.31 kg	
Aripiprazole	Komossa [107]	I	A	2	I	Mean loss of -1.37 kg or 13.5%	vs olanzapine +4.23 kg, or 36% (26 weeks)	[63]
	Kwon [108]	IV	B	2	U	Drug Naïve: 35.5% gained 2.85 kg	26 weeks: 64.5% non naïve patients gained 1.64 kg	Not ranked
Haloperidol	Alvarez-Jiminez [62]	I	A	1	I	0.01–1.4 kg or 3–10% (-0.7–0.4 kg)	10–12 wks (>9 mos)	[62], [63], [65]
		I	A	1		Drug Naïve: 2.6–3.8 kg or 22.7%	10–12 wks:3 studies, approximately 4 fold greater weight gain in drug naïve patients	Not ranked
		I	A	1		4.0–9.7 kg or 75%	3 studies, >9 mos	
Depo haloperidol	Bechelli [109]	III	B	2	U	Wt gain of ≥5 kg in 16% of patients.	Wt gain of ≥5 kg in 39% of pipothiazine palmitate patients at 8 weeks	Not ranked
Fluphenazine	Allison [65]	I	B	3	D	0.43 kg	10 weeks	[65]
Fluphenazine decanoate	Wistedt [110]	III	B	2	U	Qualitative data only	20-wk RCT: Depo Flu vs Depo HaloP: > wt inc with depo fluphenazine but NS.	Not ranked
Ziprasidone	Komossa [111]	I	A	1	I	8.3%	12 weeks vs amisulpride 17.5%	[29], [63]–[65], [75], [76]
		I	A	1		2.6%	24 wks: olanzapine 14.9%	
		I	A	1		5.8%	26 wks: olanzapine 27.4% Risperidone 13.5%	
		I	A	1		6.5%	78 wks: olanzapine 27.4% risperidone 12.3%	
		I	A	1		Studies comparing means: -1.65 kg	Studies comparing means: 24 wks: olanzapine +4.91 kg	
		I	A	1		-1.12 kg	28 wks: olanzapine +3.06 kg	
Molindone	Bagnall [112]	I	A	2	I	Molindone: 0/14 gained >4.5 kg	12,4,8 weeks: Placebo: 0/15 gained >4.5 kg. Chlorpromazine: 4/15	[65]
Perphenazine	Lieberman [29]	III	A	1	D	Mean weight loss: -0.9 kg, (12%)	78 weeks: All patients were previously on typical or atypical antipsychotics.	[29], [76]

%  =  % gaining >7% body weight. Sources of funding: D  =  direct funding from a pharmaceutical manufacturer; I  =  indirect funding (where authors had research funds) U  =  unfunded or unknown funding.

Six articles ranked the antidepressants (excluding MAOIs) (Table 4). Three articles were classified as level I with quality of evidence that was fair [66]–[68]. The most comprehensive ranking data came from one article [55]. The ranking was based on the data from drug non naïve patients. This article presented the effect of each antidepressant on weight during two treatment periods, 4–12 weeks and ≥4 months. Data from the 4–12 week interval was used to rank the antidepressants only when data from the longer time period was not available. The quality of the evidence for the change in weight was classified as good for the ≥4 month treatment period interval. However, when the duration of the treatment period was ≤12 weeks we assigned a poor quality rating to the evidence. One article was classified as level III with evidence that was fair [69] and the other article was level V with poor quality of evidence [60].

**Table 4:** Antidepressant Ranking and Effect on Body Weight (ranked from most weight gain to weight loss).

Antidepressant	Author	Study Level	Quality of Study	Quality of Evidence for the Drug	Funding Source	Quantitative Weight Change in kg. >12 weeks unless indicated	Articles used for ranking
**Weight gain**							
Paroxetine	Serretti [55]	I	B	1	U	2.73 CI 0.78 to 4.68*	[55], [66]–[68]
Mirtazapine	Serretti [55]	I	B	1	U	2.59 CI –0.23 to 5.41*	[55], [66]–[69]
Doxepin	Feighner [70]	III	B	2	U	2.73	Not ranked, placement based on quantitative data
Amitriptyline	Serretti [55]	I	B	1	U	2.24 CI 1.82 to 2.66	[55], [60], [69]
Citalopram	Serretti [55]	I	B	1	U	1.69 CI –0.97 to 4.34	[55]
Nortriptyline	Serretti [55]	I	B	1	U	1.24 CI –0.51 to 2.99	[55], [60]
Clomipramine	Serretti [55]	I	B	3	U	1.0 CI –0.44 to 2.43≤12 weeks	[55]
Desipramine	Serretti [55]	I	B	3	U	0.82 CI –0.77 to 2.42≤12 weeks	[55] [64]
Imipramine	Serretti [55]	I	B	1	U	- 0.04 CI –1.36 to 1.28*	[55] [64] Ranking based on expert panel recommendation
Duloxetine	Serretti [55]	I	B	1	U	0.71 CI –0.23 to 1.65	[55]
Escitalopram	Serretti [55]	I	B	1	U	0.65 CI –0.16 to 1.45	[55]
Trimipramine	Harris [71]	VI		3	U	Qualitative data only	Not ranked
**Minimal effect on weight**							
Venlafaxine	Serretti [55]	I	B	3	U	- 0.5 CI –0.74 to -0.27≤12 weeks *	[55]
Fluvoxamine	Serretti [55]	I	B	3	U	- 0.02 CI -0.49 to 0.45≤12 weeks	[55]
Fluvoxamine CR	Davidson [72]	III	B	1	D	Qualitative data only	Not ranked
	Westenberg [73]	III	B	1	D	Qualitative data only	Not ranked
Sertraline	Serretti [55]	I	B	1	U	- 0.12 CI –1.65 to 1.42	[55], [67]
Trazodone	Serretti [55]	I	B	3	U	- 0.2 CI -0.94 to 0.54≤12 weeks	[55]
Moclobemide	Serretti [55]	I	B	3	U	- 0.21 CI -0.30 to -0.13≤12 weeks	[55]
Fluoxetine	Serretti [55]	I	B	1	U	- 0.31 CI -1.04 to 0.43	[55], [66], [67]
Desvenlafaxine	Perry [74]	II	B	2	U	-0.8 kg, Minimal effect on weight in both short-term and long term use (12 weeks)	Not ranked
**Weight Loss**							
Bupropion	Serretti [55]	I	B	1	U	- 1.87 CI -2.37 to -1.37	[55]

Sources of funding: D  =  direct funding from a pharmaceutical manufacturer; U  =  unfunded or unknown funding *controversy in the ranking table.

Controversies in the ranking were reviewed by the expert panel and they provided their recommendations which were incorporated into the above table. Three articles [55], [67], [68] provided controversy in the ranking between paroxetine and mirtazapine. Two articles [68] and [67] both concluded that mirtazapine caused more weight gain that paroxetine. After reviewing the evidence from the three studies, the ranking from the Serretti article was selected due to the fact that the other two studies were ranked lower on our scoring system, and were of shorter duration compared to Serretti. It was also noted that the short term data from these two articles were consistent with the short term data from Serretti. In addition, although [68] and [67] were published as two separate articles, they both obtained their data from the same references.

There was agreement with the ranking of the tricyclic antidepressants based on the Serretti article except for the ranking of imipramine. Based on the clinical experience of the panel, all tricyclic antidepressants are associated with some degree of weight gain. One article [60] used to rank the antidepressants provided evidence to support the claim that imipramine causes weight gain in the long term. As a result, imipramine was ranked with, but below, the other tricyclic antidepressants.

The data from Serretti on venlafaxine was ≤12 weeks. Based on the clinical experience of the panel and the lack of long term data on venlafaxine that met our selection criteria, the panel disagreed with Serretti’s classification of venlafaxine as causing weight loss. In their experience, longer term use of venlafaxine would not result in significant weight loss and as a result it was ranked just below escitalopram as venlafaxine was observed to have minimal effect on weight in the long term.

The long term data on fluoxetine from the Serretti article would imply that fluoxetine was associated with a small weight loss. The panel considered fluoxetine as having minimal effect on weight.

Although there was no data to rank four antidepressants, doxepin, trimipramine, fluvoxamine CR and desvenlafaxine, there was quantitative and/or qualitative data available and this data was included in the ranking table 4 [70]–[74].

There was no controversy between the two articles that ranked the MAOIs [60], [61]. In the panel’s opinion, the ranking in this table was consistent with that seen in clinical practice.

Seven articles were located that met our criteria and provided data to allow us to rank the typical and atypical antipsychotics Table 3 [29], [62]–[65], [75], [76]. The ranking was based on the data from drug non naïve patients. There were a few discrepancies identified that were presented to the panel for their recommendations as five articles ranked both quetiapine and risperidone. Two articles [75] and [29] ranked quetiapine as causing more weight gain than risperidone, one article [76] provided qualitative data only stating that they both caused weight gain, one article [63] placed risperidone above quetiapine and one article [64] concluded that they were similar. After reviewing the available data the panel recommended placing quetiapine ahead of risperidone acknowledging that at this time the literature indicates the difference in weight gain between the two drugs is minimal. One article [75] also stated that the weight gain caused by olanzapine was equal to quetiapine however; the qualitative data was presented on a scale of 1–5 without providing a range for their scoring system.

We were also unable to find ranking data on drugs that were available in formulations other than oral. For the drugs that are available in formulations such as injectable that had quantitative or qualitative data, we included this data in the ranking table with the oral formulation. However the ranking of drugs in these tables only applies only to the oral formulation.

Table 5 provides the weight gain caused by typical and atypical antipsychotics and flunarizine but not ranked due to insufficient data.

**Table 5:** Weight gain caused by typical and atypical antipsychotics and flunarizine (drugs not ranked due to insufficient data).

Antipsychotic	Author	Study Level	Quality of Study	Quality of Evidence for the Drug	Funding Source	Quantitative Weight Gain	Comments
**Weight gain**							
Levopromazine	Sivaraman [113]	II	A	2	I	Qualitative data only	Similar weight gain as Chlorpromazine, 30 weeks
Trifluoperazine	Marques [114]	I	A	1	I	Qualitative data only	No difference in wt gain vs Pimozide, 6 studies only 2>12 weeks
Loxapine	Chakrabarti [115]	II	A	1	I	18.6%	At 12 weeks vs 0% in placebo
Depot flupenthixol decanoate	Johnson [116]	IV	C	3	U	62% gained 1.5 to >11 kg	6 months: 16% lost 1.5 to 4.9 kg; 22% no change; Similar to fluphenazine decanoate
Zuclopenthixol	Kumar [117]	I	A	3	I	Qualitative data only	Two studies 10 and 12 weeks: short duration and low N. No difference in weight gain compared to sulpride
Paliperidone extended release	Chwieduk [118]	I	C	2	U	1.5 kg	3–6 wk trials with 52 wk extensions. Olanzapine 3.8 kg
Paliperidone injectable	Citrome [119]	I	B	2	U	0.7 kg or 12% (mild)	Open label prior to randomization.
						6%	Double blind phase: placebo 3% since randomization.
		I	B	2		13%	Open-label extension period (relative to starting the extension phase). Lowest incidence among patients who received double-blind paliperidone – presumably had already gained the weight they were going to.
Perospirone	Okugawa [120]	III	C	3	D	Mean Weight Gain: 2.2 kg	Greater mean weight gain vs risperidone, 1.7 kg
Iloperidone	Marino [121]	I	C	2	U	4.8 kg	52 week duration: Haloperidol 3.0 kg. Weight gain may be dose related. Majority of weight gain occurs in first 6 weeks of treatment.
	Hale [122]	I	C	2	U	3.8 kg	Haloperidol 2.3 kg; 1 study of 52 weeks
Flunarizine	Bisol [123]	III	A	1	I	mean wt gain 1.2 kg or 8%	12 weeks: Haloperidol -0.8 kg or 7.4%
Asenapine	Citrome [124]	I	B	2	D	23%	vs olanzapine, 57.1% in patients with initial BMI <23
		I	B	2		9.3%	vs olanzapine, 21.9% in patients with initial BMI >27. Weight gain is not dose related.

Unless specified, %  =  % gaining >7% body weight. Sources of funding: D  =  direct funding from a pharmaceutical manufacturer; I  =  indirect funding (where authors had research funds); U  =  unfunded or unknown funding.

Among the mood stabilizers, both lithium and valproate caused weight gain (Table 6). Two studies were used to rank these two drugs. The study presented by Melvin (Level II/B) [77] described the weight gain due to both lithium and valproate as “++”. The Bowden study (Level III/B) [78] at 12 weeks ranks valproate slightly ahead of lithium (1.1 kg vs 0.2 kg). Quantitative data obtained from two different publications [79], [80] and the clinical impressions of the expert panel support the ranking of valproate slightly ahead of lithium.

**Table 6:** Change in weight caused by mood stabilizers (Ranked most to least weight gain).

Mood Stabilizer	Author	Study Level	Quality of Study	Quality of Evidence for the Drug	Funding Source	Quantitative Weight Change	Comments
**Weight Gain and Ranked [77]–[78]**							
Valproate	Leslie [80]	I	B	2	D	2.5 kg to 1.2 kg	At 12 weeks and 47 weeks respectively.
Valproate Extended Release	Smith [125]	I	B	3	D	19/103	9 studies (2–6 weeks x 5; 1–12 weeks x 4). Compared to delayed release caused less weight gain 29/103. (not ranked)
Lithium	Bowden [79]	III	A	1	D	1.1 kg in lean patients	A randomized, double-blind, placebo-controlled study at 52 weeks. 6.1 kg in obese patients.
**Weight Neutral**							
Carbamazepine Extended Release	Ketter [126]	IV	B	2	D	Qualitative data only	26 weeks. Based on one study.
Carbamazepine	Melvin [77]	II	B	3	I	Qualitative data only	Study duration not provided.
Oxcarbazine	Reinstein [127]	III	C	2	D	Qualitative data only	10 weeks
Lamotrigine	Bowden [79]	III	A	1	D	- 0.5 kg in lean patients	A randomized, double-blind, placebo-controlled study at 52 weeks. -4.2 kg in obese patients.
**Weight Loss**							
Topiramate	Stoffers [128]	I	A	3	I	Qualitative data only	3 studies all <12 weeks demonstrate weight loss vs placebo. Many studies have used topiramate for weight loss however, few were done in psychiatric illness.

Sources of funding: D  =  direct funding from a pharmaceutical manufacturer; I  =  indirect funding (where authors had research funds).

### Anxiolytics

Qualifying papers were found for five benzodiazepines and buspirone (Table 7). All of the anxiolytics were weight neutral. Unfortunately, the highest level of evidence was III and all of the data was qualitative only. There was no information for the previous drug status of the patients included in these studies.

**Table 7:** Change in weight caused by anxiolytics (Not ranked).

Anxiolytics	Author	Study Level	Quality of Study	Quality of Evidence for the Drug	Funding Source	Quantitative Weight Change	Comments
**Benzodiazepines – Weight Neutral**							
Nitrazepam	Oswald [129]	III	B	2	U	Qualitative data only	5 months
Chlordiazepoxide	Bjertnaes [130]	VI	NA	3	U	Qualitative data only	6 weeks
Lorazepam	Smits [131]	IV	A	2	U	Qualitative data only	Cross sectional
Diazepam	Smits [131]	IV	A	2	U	Qualitative data only	Cross sectional
Oxazepam	Smits[131]	IV	A	2	U	Qualitative data only	Cross sectional
**Serotonin 1A Agonists – Weight Neutral**							
Buspirone	Yuanguang [132]	VI	NA	NA	U	Qualitative data only	4 weeks

U  =  unfunded or unknown funding; NA  =  not able to assess.

## Discussion

In this review we used a predefined strategy to search for the available evidence on the ability of psychotropics to induce changes in body weight. The articles were selected based on a hierarchical level of evidence and were subsequently evaluated using AMSTAR for systematic reviews and SIGN 50 for controlled trials. The best evidence available was presented. We restricted our search to subjects with psychiatric disease since this review is intended as a resource to help choose psychotropics for psychiatric illness according to risk of weight gain.

Although most antipsychotics were found to be associated with weight gain, there are inherent difficulties in quantifying this weight. Many trials did not account for weight gain among the reported side-effects, some reported change in mean body weight, and some reported the percentage who gained more than 7% of their initial body weight. Many studies did not consider drug dosages or parameters for drug adherence, gender, and pharmacogenetics. Most studies had high dropout rates. There are factors that would result in significant underestimations of weight gain potential. These include studies of short duration, the use of last observation carried forward to handle data from study dropouts, previous drug use that would cause weight gain, and industry sponsorship.

Since the treatment of psychiatric illness often takes months or years, and because it takes time for weight gain to develop, we selected articles with study duration of 12 weeks or longer. Unfortunately, many of the randomized clinical trials were of short duration and thus were not able to provide sufficient information about the full impact of the drug on body weight. Kinon [81] and Tran [82] reported on the time course of weight gain with olanzapine; they showed continued weight gain up to 39 and 22 weeks.

Recovery from the psychiatric illness itself may influence study outcome. This may be a more important factor in the treatment of depression than of other psychiatric disorders [83]. Also, measures that patients take to offset weight gain are rarely discussed but may influence the degree to which a patient gains weight.

The effect of drug dosage on weight gain has been reviewed [84], but it is rarely discussed in reviews. We minimized this effect by verifying that all studies and reviews also had efficacy as an outcome measure.

We found only two studies that addressed the issue of drug adherence by determining plasma drug levels in the study subjects [85], [86]. Genetic and gender differences may also be significant factors affecting a patient’s side-effect response to these drugs [87]. Pharmacogenetics approaches may offer the possibility of identifying patient-specific biomarkers for predicting the risk of these side effects [88]. A retrospective chart review [89] indicates that women and those with a greater initial BMI are more susceptible to weight gain [87], for example, obese patients given lithium gained more weight on lithium compared with lean patients [79]. There were high drop-out rates in many of the studies. In one study 74% of the patients discontinued the study medication within 18 months. The Last Observation Carried Forward (LOCF) method used in many studies for dealing with dropouts is likely to underestimate drug-associated weight gain [90].

Many studies have confounding variables that have contributed to the underestimation of drug-induced weight gain. Weight gain differs between those with previous psychotropic treatments and those previously unexposed to psychotropics. In patients who are not drug-naïve, weight gain can be affected by the previous drug as well as the study drug. For example, studying the weight gains with long-acting risperidone in patients who had been switched from other antipsychotics, Lindenmayer [91] found an overall mean weight gain of 0.4 kg over 12 weeks. The same study found a gain of 1.4 kg in patients who had been on haloperidol and of 0.3 kg in those who had been on quetiapine, and a loss of 0.5 kg in those on olanzapine. This shows that absolute weight gain is underestimated in studies that include patients who are not drug-naïve. Weight gain was three to four times greater in studies that included individuals with limited previous exposure to antipsychotic drugs [62].

Approximately one third of the studies presented in the tables were directly funded by pharmaceutical manufacturers. This number may be underestimated because many of the studies did not declare their source of funding. Two systematic reviews, Sismondo, and Ahmer conclude that pharmaceutical company sponsorship is strongly associated with results that favour the sponsors’ interests [92], [93]. In studying “wish bias” in antidepressant drug trials, Barbui found that fluoxetine was favoured in clinical trials when fluoxetine was the experimental agent, and that comparator antidepressants were favoured in trials using fluoxetine as the reference agent [94]. In a report with a noteworthy title (“Why Olanzapine beats Risperidone, Risperidone beats Quetiapine and Quetiapine beats Olanzapine”) Heres et al come to the same conclusion and suggest ways in which potential sources of bias can be addressed by study initiators, peer reviewers and readers [95]. However, in a secondary analysis of a systematic review, Gartelhner found that the effect of study sponsorship on a systematically evaluated body of evidence of head-to-head trials was modest and perhaps not clinically significant [96].

We saw an urgent need for a clinical tool to allow choice of psychotropic drugs with respect to weight change. A full systematic review was beyond the scope of our resources, we therefore developed this hierarchical approach. The biggest challenge in conducting this systematic synthesis was the analysis of very heterogeneous study designs. While we have done our best to summarize the extremely large amount of published literature, we caution the user about the limitations of this analysis. These limitations include drug dosage, variation in reporting of weight gains, use of drug naïve versus non-drug naïve patients, mono-therapy vs. combination therapy, duration of treatment, psychiatric diagnosis, baseline patient characteristics such as age, gender, BMI, genetic factors, recovery from the underlying condition and concurrent weight treatments during the study.

The findings of this review highlight the need for the development of psychotropics that are not associated with weight gain. As well, a better understanding of the pharmacogenetics of psychotropic drug response might help select psychotropics for individuals so that weight gain is minimized. It is important to consider methods for minimizing the impact of weight gain induced by psychotropic drugs. Choices must be made on a case-by-case basis, with careful consideration of issues of weight, therapeutic efficacy, and other relevant factors discussed in this paper in order to minimize the impact of weight gain with psychotropic medications.

Further research is needed to determine actual weight gain for all psychotropics in drug naïve patients for sufficient lengths of time to determine the full impact of the weight gain and co-morbidities of this weight gain. These studies should be done at arms-length from industry funding and reported in both mean weight change and percent who gain more than 7% of initial body weight.

## Supporting Information

Figure S1 **PRISMA 2009 Flow Diagram.**
(TIF)

Appendix S1 Ovid Medline search strategy.
(DOCX)

Appendix S2 PsycINFO search strategy.
(DOCX)

Appendix S3 CCTR, CDSR (coch), Dare Search Strategy.
(DOCX)

Appendix S4 Embase search strategy.
(DOCX)
