# Supplementary figures and images for: Changes in Body Weight and Psychotropic Drugs: A Systematic Synthesis of the Literature
Source: PLoS One. 2012 Jun 15;7(6):e36889. doi: 10.1371/journal.pone.0036889 (PMC3376099; doi:10.1371/journal.pone.0036889)

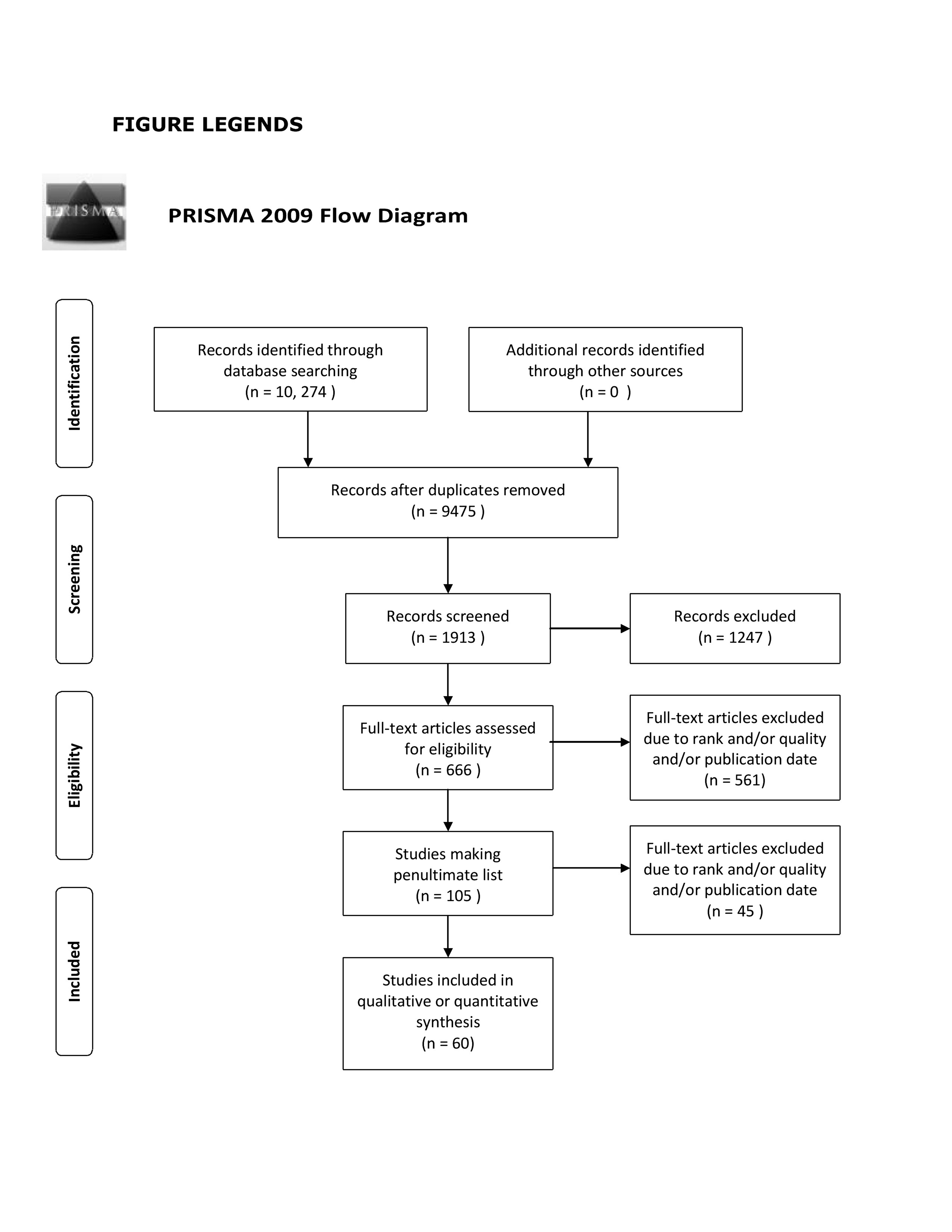

Supplement: Figure S1 — PRISMA 2009 Flow Diagram. (TIF) [file pone.0036889.s001.tif]
