# Supplementary material for: Changes in Body Weight and Psychotropic Drugs: A Systematic Synthesis of the Literature
Source: PLoS One. 2012 Jun 15;7(6):e36889. doi: 10.1371/journal.pone.0036889 (PMC3376099; doi:10.1371/journal.pone.0036889)
Supplement: Appendix S1 — Ovid Medline search strategy. (DOCX) [file pone.0036889.s002.docx]

**Appendix S1: Ovid Medline search strategy**

1 Weight Gain/ (14192)

2 Body Weight/ (132480)

3 (weight adj2 gain$).tw. (28964)

4 (weight adj2 (increas$ or change)).tw. (15282)

5 Obesity/ (73152)

6 Obesity, Morbid/ (5145)

7 Body Mass Index/ (35460)

8 or/1-7 (248626)

9 anxiolytic$.tw. (7058)

10 (benzodiazepine$ or chlordiazepoxide or librium or diazepam or valium or nitrazepam or mogadon or lormetazepam or loramet).mp. (47747)

11 (buspirone or buspar).mp. (2175)

12 antidepressive agents, tricyclic/ (7984)

13 (amitriptyline or elavil or endep or clomipramine or anafranil or desipramine or norpramin or doxepin or sinequan or adapin or imipramine or tofranil or nortriptyline or aventyl or protriptyline or triptil or vivactil or trimipramine or surmontil).mp. (25422)

14 serotonin uptake inhibitors/ or ssri$.tw. (11380)

15 (citalopram or celexa or fluoxetine or prozac or fluvoxamine or luvox or paroxetine or paxil or sertraline or zoloft).mp. (14682)

16 exp antimanic agents/ or mood stabilizer$.tw. (19651)

17 (lithium or valproate or valproic acid or depakene or carbamazepine or tegretol or gabapentin or neurontin or lamotrigine or lamictal or topiramate or topamax).mp. (46762)

18 antipsychotic agents/ or (antipsychotics$ or neuroleptic agent$ or neuroleptic drug$).tw. (30912)

19 (chlorpromazine or largactil or thorzine or fluphenazine or moditen or prolixin or haloperidol or haldol or perphenazine or trilafon or sulpiride or dogmatil or thioridazine or mellaril or pipothiazine or piportil).mp. (41006)

20 (clozapine or clozaril or olanzapine or zyprexa or quetiapine or seroquel or risperidone or risperdal or ziprasidone or geodon or aripiprazole or abilify or abilitat or amisulpride or amis or solian or zotepine or zot).mp. (13739)

21 (Serotonin norepinephrine reuptake inhibitor$ or snri$).tw. (298)

22 (venlafaxine or effexor or duloxetine or cymbalta or reboxetine or edronax or vestra or iloperidone or zomaril or maprotiline or ludiomil).tw. (3047)

23 (Norepinephrine dopamine reuptake inhibitor$ or ndri$).tw. (12)

24 (Bupropion or wellbutrin or amfebutamone).mp. (1981)

25 (serotonin antagonist$ or trazodone or desyrel or mirtazepine or remeron).mp. (14200)

26 (maoi$ or monoamine oxidase inhibitor$ or isocarboxazid or marplan or phenelzine or nardil or tranylcypromine or parnate or moclobemide or mannerix).mp. (11992)

27 or/9-27 (220934)

28 clinical trial.pt. (441236)

29 randomized controlled trial.pt. (242307)

30 random$.tw. (385510)

31 placebo$.mp. (117846)

32 Double blind method/ or Single blind method/ (104130)

33 ((singl$ or doubl$ or treb$ or tripl$) adj2 (blind$ or mask$)).tw. (90502)

34 case-control studies/ or retrospective studies/ (361599)

35 (retrospective or longitudinal).tw. (209141)

36 cohort studies/ or exp longitudinal studies/ or follow-up studies/ (629797)

37 ((cohort or follow up or observational) adj (study or studies)).tw. (71371)

38 meta-analysis.pt. (16501)

39 (medline or systematic review$).tw. (30823)

40 (meta-analys$ or metaanalys$).tw. (18633)

41 guideline.pt. (14291)

42 or/29-42 (1606305)

43 8 and 28 (4851)

44 43 and 44 (1655)

45 (animals not humans).sh. (3180108)

46 45 not 46 (1601)

47 limit 47 to english language (1540)
