# Supplementary material for: Changes in Body Weight and Psychotropic Drugs: A Systematic Synthesis of the Literature
Source: PLoS One. 2012 Jun 15;7(6):e36889. doi: 10.1371/journal.pone.0036889 (PMC3376099; doi:10.1371/journal.pone.0036889)
Supplement: Appendix S2 — PsycINFO search strategy. (DOCX) [file pone.0036889.s003.docx]

**Appendix S2: PsycINFO search strategy**

1 body weight/ (6951)

2 (weight adj2 gain$).tw. (4253)

3 (weight adj2 (increas$ or change)).tw. (1627)

4 obesity/ (6658)

5 or/1-4 (15322)

6 anxiolytic$.tw. (4028)

7 (benzodiazepine$ or chlordiazepoxide or librium or diazepam or valium or nitrazepam or mogadon or lormetazepam or loramet).mp. (11115)

8 (buspirone or buspar).mp. (1144)

9 tricyclic antidepressant drugs/ (457)

10 (amitriptyline or elavil or endep or clomipramine or anafranil or desipramine or norpramin or doxepin or sinequan or adapin or imipramine or tofranil or nortriptyline or aventyl or protriptyline or triptil or vivactil or trimipramine or surmontil).mp. (8426)

11 (serotonin reuptake inhibitor$ or ssri$).mp. (5273)

12 (citalopram or celexa or fluoxetine or prozac or fluvoxamine or luvox or paroxetine or paxil or sertraline or zoloft).mp. (8173)

13 (mood stabilizer$ or lithium or valproate or valproic acid or depakene or carbamazepine or tegretol or gabapentin or neurontin or lamotrigine or lamictal or topiramate or topamax).mp. (11613)

14 neuroleptic drugs/ or (antipsychotic$ or neuroleptic agent$ or neuroleptic drug$).tw. (17769)

15 (chlorpromazine or largactil or thorzine or fluphenazine or moditen or prolixin or haloperidol or haldo or perphenazine or trilafon or sulpiride or dogmatil or thioridazine or mellaril or pipothiazine or piportil).mp. (11147)

16 (clozapine or clozaril or olanzapine or zyprexa or quetiapine or seroquel or risperidone or risperdal or ziprasidone or geodon or aripiprazole or abilify or abilitat or amisulpride or amis or solian or zotepine or zot).mp. (9306)

17 (serotonin norepinephrine reuptake inhibitor$ or snri$).mp. (235)

18 (venlafaxine or effexor or duloxetine or cymbalta or reboxetine or edronax or vestra).mp. (1401)

19 (norepinephrine dopamine reuptake inhibitor$ or ndri$).tw. (13)

20 (amfebutamone or bupropion or wellbutrin).mp. (960)

21 (serotonin antagonist$ or trazodone or desyrel or mirtazepine or remeron).mp. (2998)

22 (maoi$ or monoamine oxidase inhibitor$ or isocarboxazid or marplan or phenelzine or nardil or tranylcypromine or parnate or moclobemide or mannerix).mp. (2602)

23 or/6-23 (70737)

24 5 and 24 (1722)

25 random$.tw. (68610)

26 double-blind.tw. (11156)

27 control$.tw. (290361)

28 effectiveness.tw. (64581)

29 exp treatment/ (381190)

30 (meta-analys$ or metanalys$ or systematic review$).tw. (9254)

31 search.tw. (26185)

32 (followup study or longitudinal study or retrospective study or systematic review or treatment outcome or randomized clinical trial).md. (72369)

33 longitudinal studies/ or prospective studies/ or followup studies/ or retrospective studies/ (26967)

34 or/26-34 (757543)

35 25 and 35 (1461)

36 limit 36 to human (1344)

37 limit 37 to english language (1272)
