# Supplementary material for: Changes in Body Weight and Psychotropic Drugs: A Systematic Synthesis of the Literature
Source: PLoS One. 2012 Jun 15;7(6):e36889. doi: 10.1371/journal.pone.0036889 (PMC3376099; doi:10.1371/journal.pone.0036889)
Supplement: Appendix S4 — Embase search strategy. (DOCX) [file pone.0036889.s005.docx]

**Appendix S4: Embase search strategy**

1 *Weight Gain/ (2744)

2 Body Weight/ (56813)

3 (weight adj2 gain$).tw. (23080)

4 (weight adj2 (increas$ or change)).tw. (12439)

5 *OBESITY/ (37428)

6 or/1-5 (111957)

7 anxiolytic agent/ or anxiolytic$.tw. (11825)

8 (benzodiazepine$ or chlordiazepoxide or librium or diazepam or valium or nitrazepam or mogadon or lormetazepam or loramet).mp. (78539)

9 (buspirone or buspar).mp. (5920)

10 tricyclic antidepressant agent/ (18566)

11 (amitriptyline or elavil or endep or clomipramine or anafranil or desipramine or norpramin or doxepin or sinequan or adapin or imipramine or tofranil or nortriptyline or aventyl or vivactil or protriptyline or triptil or trimipramine).mp. (49971)

12 serotonin uptake inhibitor/ or ssri$.tw. (21236)

13 (citalopram or celexa or fluoxetine or prozac or fluvoxamine or luvox or paroxetine or paxil or sertraline or zoloft).mp. (36416)

14 atypical antipsychotic agent/ or amisulpride/ or aripiprazole/ or clozapine/ or olanzapine/ or quetiapine/ or risperidone/ or ziprasidone/ or zotepine/ (27728)

15 (clozapine or clozaril or olanzapine or zyprexa or quetiapine or seroquel or risperidone or risperdal or ziprasidone or geodon or aripiprazole or abilify or abilitat or amisulpride or amis or solian or zotepine or zot).mp. (27448)

16 neuroleptic agent/ or (antipsychotic$ or neuroleptic drug$ or neuroleptic agent$).tw. (39899)

17 (chlorpromazine or largactil or thorzine or fluphenazine or moditen or prolixin or haloperidol or haldol or perphenazine or trilafon or sulpiride or dogmatil or thioridazine or mellaril or pipothiazine or piportil).mp. (57277)

18 (mood stabilizer$ or lithium or valproate or valproic acid or depakene or carbamazepine or tegretol or gabapentin or neurontin or lamotrigine or lamictal or topiramate or topamax).mp. (84252)

19 (serotonin norepinephrine reuptake inhibitor$ or snri$).tw. (369)

20 Noradrenalin Uptake Inhibitor/ or ndri$.tw. (1775)

21 (venlafaxine or effexor or duloxetine or cymbalta or reboxetine or edronax or vestra or iloperidone or zomaril or maprotiline or ludiomil).mp. (12815)

22 (amfebutamone or bupropion or wellbutrin).mp. (6988)

23 (serotonin antagonist$ or trazodone or desyrel or mirtazepine or remeron).mp. (13344)

24 (maoi$ or monoamine oxidase inhibitor$ or isocarboxazid or marplan or phenelzine or nardil or tranylcypromine or parnate or moclobemide or mannerix).mp. (15512)

25 or/7-25 (286221)

26 6 and 26 (4495)

27 Randomized controlled trial/ (148552)

28 random$.tw. (348780)

29 PLACEBO/ (103924)

30 placebo$.tw. (101112)

31 clinical trial$.mp. (530595)

32 double-blind method/ or single-blind method/ (72770)

33 ((singl$ or doubl$ or trebl$ or tripl$) adj2 (blind or mask$)).ti,ab. (83182)

34 "Systematic Review"/ or systematic review$.tw. (26154)

35 longitudinal study/ or case control study/ or Retrospective Study/ or cohort analysis/ or practice guideline/ or observational study/ (237246)

36 ((Cohort or case control or longitudinal or retrospective or follow up or observational) adj (study or studies)).tw. (149961)

37 meta analysis/ (31878)

38 (metaanaly$ or metaanalys$).tw. (1002)

39 or/28-39 (1069223)

40 27 and 40 (1902)

41 limit 41 to human (1835)

42 limit 42 to english language (1725)
